# Validation of dietary whey protein substrate challenge absorption test as an indicator of proteolysis

**DOI:** 10.1371/journal.pone.0323730

**Published:** 2025-05-19

**Authors:** Kateryna Pierzynowska, Stefan Pierzynowski, Kamil Zaworski, Robert Gallotto, Meghana Sathe, Steven D. Freedman, Drucy Borowitz

**Affiliations:** 1 Department of Biology, Lund University, Lund, Sweden; 2 Department of Animal Physiology, The Kielanowski Institute of Animal Physiology and Nutrition, Polish Academy of Sciences, Jabłonna, Poland; 3 Anara AB, Trelleborg, Sweden; 4 Anagram Therapeutics, Inc., Natick, Massachusetts, United State of America; 5 Department of Medical Biology, Institute of Rural Health, Lublin, Poland; 6 Division of Pediatric Gastroenterology, Hepatology and Nutrition, University of Texas Southwestern/Children’s Health, Dallas, Texas, United State of America; 7 Beth Israel Deaconess Medical Center, Division of Gastroenterology, Boston, Massachusetts, United State of America; 8 Department of Pediatrics, Jacobs School of Medicine and Biomedical Sciences, University at Buffalo, Buffalo, New York, United State of America; North-Caucasus Federal University, RUSSIAN FEDERATION

## Abstract

**Introduction:**

Current methods to measure the effectiveness of pancreatic protease activity are inadequate. We explored the measurement of peptide-derived amino acids following ingestion of dietary whey substrate as a sensitive test of exogenous protease activity in exocrine pancreatic insufficient (EPI) pigs.

**Methods:**

We studied the activity of aspergillus protease given in combination with a novel lipase and fungal amylase, as well as commercially available pancrelipase in EPI pigs. After a high-fat diet plus a standardized dietary whey substrate, blood was withdrawn at intervals and was analyzed for amine groups using a modified ninhydrin reaction. Plasma peptide-derived amino acids were calculated.

**Results:**

The AUC_6_ peptide-derived amino acid concentration was significantly increased in response to aspergillus protease as follows: 50 mg dose (137% increase; p = 0.05), 75 mg dose (154% increase; p = 0.008) compared to no enzyme. The AUC_6_ for peptide-derived amino acids after aspergillus protease increased by 133% for the 50 mg dose (p = 0.0044), by 171% for the 75 mg dose (p = 0.0002), and by 113% with 600 mg pancrelipase (p < 0.0001) when compared to no enzyme. Administration of 75 mg of aspergillus protease led to significantly higher peptide-derived amino acid AUC_6_ and Cmax when compared to 600 mg pancrelipase (p = 0.0419 and 0.0103, respectively).

**Conclusion:**

In EPI pigs, measurement of peptide-derived amino acids following a meal with whey substrate differentiates the activity of aspergillus protease compared to no enzyme and the 75 mg dose was superior to pancrelipase. The evidence presented here in EPI pigs demonstrates that the whey substrate absorption challenge test reflects the proteolytic activity of different doses of exogenous pancreatic proteases.

## 1. Introduction

Digestion of proteins begins in the stomach through the action of acid and pepsin, but pancreatic proteases are needed to complete this process. It has long been recognized that people with exocrine pancreatic insufficiency (EPI) have azotorrhea as well as steatorrhea and that the two are correlated [1,2]. EPI is treated with exogenous oral pancreatic enzyme replacement therapy (PERT) containing lipase, protease and amylase taken before every meal and snack.

Calculation of the coefficient of fat absorption, a ratio of ingested to excreted fat, has been used to determine performance of PERT because of the emphasis on lipase activity and control of steatorrhea. The coefficient of nitrogen absorption (CNA) is performed using a similar concept (protein in: protein out), although CNA also measures the digestion and absorption of endogenous proteins secreted into the intestine. CNA has not been used to determine optimal protease dose.

The most widely used measure of nutritional status in people with CF over age 2 years is body mass index (BMI) [3,4]. Various methods have been used to assess the fat-free mass in people with CF and suggest that BMI likely underestimates the deficiency of fat-free mass [5]. Isotopic methods have been used in healthy adults to assess whole body protein turnover [6,7] and include but are not specific to absorption. In addition to decreased availability of protein as a substrate, people with CF and EPI may also have excessive catabolism due to respiratory disease and inflammation [8]. Thus, neither measures of excreted nitrogen nor whole body composition can be used to evaluate the effect of different doses of protease to treat the maldigestion and malabsorption of protein in people with EPI.

Most studies of protein digestion and absorption employ oral liquid substrates and not food. For example, the provision of essential amino acid mixtures can increase amino acid absorption and protein synthesis in people with CF [9]. A recent method [10] assessed protein digestibility but not protease dose. It is estimated that the appearance of amino acids in the blood only corresponds to between 10–20% of protein digestion and absorption [11,12]. The remainder of dietary protein appears in the blood in the form of oligopeptides, whose functional actions [12–14] are likely to make them a more relevant measure of protein digestion than excreted nitrogen. Di- and tripeptides pass from the gut lumen to the blood via PepT1 H^+^/peptide co-transporters [15], found on enterocytes. Currently, there is no reliable method for the measurement of oligopeptide absorption. We modified a ninhydrin method, which is widely used to analyze and characterize amino acids, peptides, and proteins [16], in biomedical, clinical, food, forensic, histochemical, microbiological, nutritional, and plant studies, for the measurement of peptide-derived amino acids. This method excludes larger plasma proteins such as albumin and fibrinogen. We hypothesized that the post-prandial appearance of peptides below 10 kDa in size in circulation would be a reliable marker for the estimation of protease activity.

We sought to study a whey substrate absorption challenge test (SACT) in an EPI pig model to measure the absorption of oligopeptides as a reflection of protease activity. The development of this pharmacokinetic-like test was part of the process to clarify the activity of each component of a novel PERT. Our aim was to have a test that would enable dose-ranging studies of the protease component of a drug product to treat EPI that would contain lipase, protease and amylase. In addition, we aimed to determine the postprandial changes in concentration, area under the curve (AUC), mean over 6 hours, and concentration peak (Cmax) at time points that would help to define physiological effects of proteolysis and absorption.

## 2. Materials and methods

This study with an adaptive design followed principles of Good Laboratory Practice as defined by the Organization for Economic Co-operation and Development as applicable. Procedures involving the care and the use of animals in this study were reviewed and approved by the Second Warsaw Local Ethics Committee for Animal Experimentation in Warsaw, Poland, permission number WAW2/025/2022. During the study, the care and use of animals was conducted in accordance with the principles outlined in the current Guide to the Care and Use of Experimental Animals.

### 2.1. Exocrine pancreatic insufficient (EPI) pig model

The EPI pig is an established surgical model commonly used to study the uptake of macronutrients and to evaluate different preparations of orally administered pancreatic enzymes [17–19]. Pigs were sedated using azaperone (Stresnil, ELANCO, Warsaw, Poland) at 4 mg/kg body weight (bw), given intramuscularly (i.m.). The pigs were then anaesthetised using 0.5–1.5% air mixture of Fluothane (Zeneca, Gothenburg, Sweden) and O_2_ as a carrier gas, at approximately 0.5–1 l/min in a closed circuit respiratory system (Komesaroff Medical Developments, Melbourne, Australia). Surgical anaesthesia was indicated by the lack of a corneal reflex. Postoperative pain was prevented by the administration of buprenorphine (Temgesic®, Roche, Warsaw, Poland, 0.01 mg/kg bw, i.m.). Ampicillin (Ampicillin TZF, Polfa Tarchomin, Tarchomin, Polska) was administrated by intravenous (i.v.) injection (15 mg/kg bw) for three days after surgery. At the end of the study, all pigs were euthanized by an i.v. injection of an overdose of pentobarbital sodium (Euthanimal, Alfasan, Leżajsk, Polen, 100 mg/kg bw).

After duct ligation, young pigs develop steatorrhea and show impaired growth. Total EPI dramatically reduces the levels of digestive enzymes in the duodenum and local pH is reduced by approximately 1–2 units, causing a reduction in fat digestion and absorption. EPI pigs, weighing around 10–12 kg upon commencement of research studies, have food and energy requirements which correspond to that of children between 6 and 12 years old.

EPI pigs were individually housed in collection cages equipped with a dry feeding trough, a drinking nipple, and a constant heating lamp. All pigs were allowed to move freely within their cages, had visual contact with one another, and were weighed once a week. During the study period, pigs were fed an amount equivalent to 4% of their body weight daily with 1% given at the morning meal and approximately 3% at the afternoon meal. Meals were solid pig chow containing 3% fat, 21% protein, 73% carbohydrates and 3% minerals/vitamins (Morawski Plant, Kcynia, Poland). Following the surgery, and beginning on Day -7, they were fed a high fat diet containing 20% fat, 17.5% protein, 57.3% carbohydrates and 5.2% minerals/vitamins (HFD 20, Kcynia, Morawski Plant, Poland). The morning meal of the SACT mimicked a standard high-fat meal for a person with CF (fat 36–40%, protein 15–20%, carbohydrate 40–49%).

The first experimental period explored different doses of aspergillus protease. This lasted for two weeks and was comprised of five ‘blocks’ of 3 days during which a SACT with various enzyme doses were tested on Day One, with a two-day washout period to follow. The two-day no enzyme test period was between the first two and the second two protease dose periods. The second experimental period was a comparison of the two highest aspergillus protease doses and a standard dose of pancrelipase to no enzyme and had four experimental blocks. Pigs were divided into two groups with block randomization based on their degree of steatorrhea and body weight. These groups only differed in the sequence of administration of the enzyme doses to minimize possible period effects. Each pig had eight 3–5 mL blood samples withdrawn over the 24-hour SACT period at the following time points: 0, 1, 2, 4, 6, 8, 12, and 24 hours.

### 2.2. Peptide analysis

The levels of free, total and peptide-derived amino acid equivalents were estimated by a modified ninhydrin method adapted for the microplate reader [20]. Ninhydrin reacts with free alpha-amino acids to produce a deep purple or blue color, known as Ruhemann’s purple, which can be measured spectrophotometrically at a wavelength of 570 nm [21]. Centrifugal filters of 10 kDa are employed to separate free amino acids and peptides from plasma proteins. The hydrolysis of permeate by exposure to acetic buffer for 1 h at 100°C releases the amino acids from peptides, enabling measurement of the nitrogen contributed by the amino acids (=total amino acids). Subtraction of free amino acids from total amino acids provides a quantification of peptide-derived amino acids using the following equation: 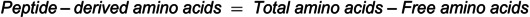


### 2.3. Test articles

We used a commercially available protease produced by *Aspergillus melleus* (EC 3.4.21.63; Seaprose-S, Amano Enzymes, Nagoya, Japan) which has broad substrate specificity for dietary proteins, hydrolyzing peptide bonds nonspecifically into peptides and amino acids. Pigs simultaneously received a recombinant I.2 class lipase variant and *Aspergillus oryzae* α-amylase in proportions as shown in Table 1. All three enzymes were combined as a novel non-porcine pancreatic enzyme replacement product (ANG003, formerly SNSP003). Two mid-level protease dose cohorts were used as this experiment was also studying variable dosing of lipase and amylase (Table 1). The 50 mg and 75 mg protease doses were compared to results using commercially available porcine-derived pancrelipase (Kreon® 25 000, Mylan Healthcare, Warsaw, Poland). The pancrelipase was provided as 50,000 lipase units per meal, at a per kg dose ~50% higher than maximum recommended dose for people with CF and EPI. Pancrelipase, a biologic extract that contains lipase, protease and amylase in the approximate proportions of 1: 3: 5 [22], is usually measured in international units: 50,000 IU is equivalent to 600 mg of pancrelipase.

**Table 1:** Proportions of experimental enzymes provided to EPI pigs.

Dose	Protease (mg)	Lipase (mg)	Amylase (mg)
1	25	20	40
2	50	80	120
3	75	120	160

The substrate was 10 gm of an unflavored whey protein isolate (California Gold Nutrition 100% whey protein isolate; www.californiagoldnutrition.com).

### 2.4. Statistical analysis

Statistical analysis was performed on the data generated from this study using the Brown-Forsythe and Welch ANOVA when evaluating multiple comparisons for normally distributed datasets or Kruskall-Wallis test with uncorrected Dunn’s test for multiple comparisons when data was not normally distributed (GraphPad Prism 8.4, USA). Differences were considered significant if *p* ≤ 0.05; differences were considered as a trend when *p* ≤ 0.1; data with Gaussian distribution are expressed as mean ± standard deviation (± SD), data with non-Gaussian distribution are expressed as median ± interquartile range (± IQR). The data was tested for normal distribution using the Shapiro-Wilk normality test. Outliers within data sets were identified using the ROUT method of regression, using (Q = 0.5%).

## 3. Results

The first experiment was conducted on 12 EPI pigs and the second experiment was conducted on 6 EPI pigs that weighed ~13.5 kg (range 10–16 kg).

### 3.1. Free amino acids

**Fig 1:**
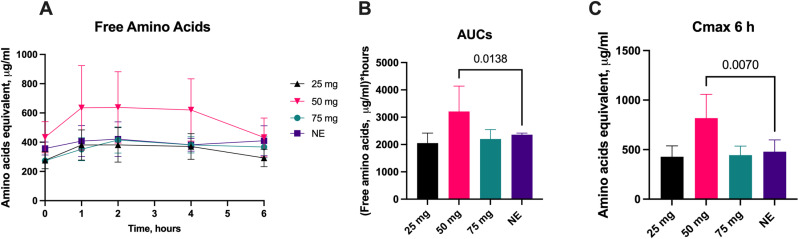
Plasma Free Amino Acid Changes with Escalating Protease Doses. Change in plasma free amino acids concentrations (A), 6-hour area under the curve (AUC_6_) (B), mean concentration over time (C) and C_max_ over 6-hours (D) following administration of various aspergillus protease doses compared to no enzyme (NE). Data on AUC_6_ is presented as median ±IQR, other data is presented as mean ±SD. Differences were considered significant if p ≤ 0.05; differences were considered as a trend when p ≤ 0.1.

The concentration of absorbed free amino acids in plasma of EPI pigs was relatively small (200–500 ug/mL. The 50 mg aspergillus protease dose significantly increased the calculated AUC_6_ plasma free amino acid concentration (Fig 1B) compared to no enzyme and significantly increased the mean plasma free amino acid concentration over the period of 6h compared to no enzyme (Fig 1C). There was a significant increase in C_max_ compared to no enzyme for the 50 mg dose (Fig 1D).

### 3.2. Total amino acids

**Fig 2:**
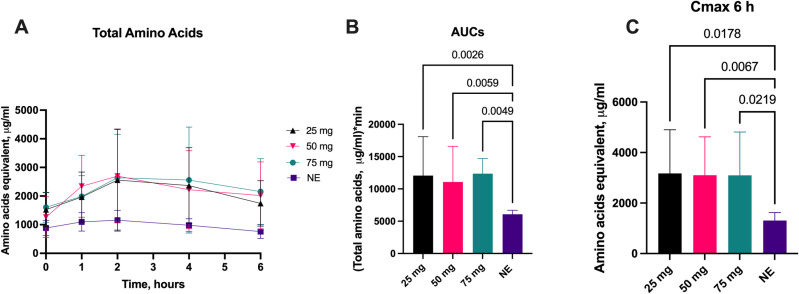
Plasma Total Amino Acid Changes with Escalating Protease Doses. Change**s** in plasma total amino acids concentrations (A), 6-hour area under the curve (AUC_6_) (B), mean concentration over time (C) and C_max_ over 6-hours (D) following administration of various aspergillus protease doses compared to no enzyme (NE). Data on AUC_6_ is presented as median ±IQR, other data is presented as mean ±SD. Differences were considered significant if p ≤ 0.05; differences were considered as a trend when p ≤ 0.1.

The effect of different doses of aspergillus protease (25 mg, 50 mg, and 75 mg) for total amino acid concentration absorbed over 6-hours when given with a fixed high-fat meal and substrate challenge is shown in Fig 2A. All doses significantly increased the calculated AUC_6_ plasma total amino acid concentration (Fig 2B) compared to no enzyme and significantly increased the mean plasma total amino acid concentration over the period of 6h compared to no enzyme (Fig 2C). There was a significant increase in C_max_ compared to no enzyme for all protease doses (Fig 2D).

### 3.3. Peptide-derived amino acids

**Fig 3:**
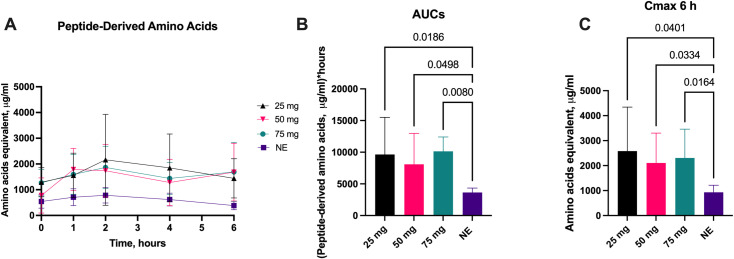
Plasma Peptide-Derived Amino Acid Changes with Escalating Protease Doses. Change**s** in plasma peptide-derived amino acids concentrations (A), 6-hour area under the curve (AUC_6_) (B), mean concentration over time (C) and C_max_ over 6-hours (D) following administration of various aspergillus protease doses compared to no enzyme (NE). Data on AUC_6_ is presented as median ±IQR, other data is presented as mean ±SD. Differences were considered significant if p ≤ 0.05; differences were considered as a trend when p ≤ 0.1.

The effect of different doses of aspergillus protease (25 mg, 50 mg, and 75 mg) for peptide-derived amino acid concentration over 6-hours when given with a fixed CF meal and substrate challenge is shown in Fig 3A. All protease doses significantly increased the calculated AUC_6_ plasma peptide-derived amino acid concentration (Fig 3B) with the 50 mg dose leading to a 137% increase (p = 0.05) and the 75 mg dose leading to a 154% increase (p = 0.008) compared to no enzyme. In all dose cohorts, the mean plasma peptide-derived amino acids concentration increased significantly over the period of 6h compared to no enzyme (Fig 3C). The protease doses of 25 mg, 50 mg, and 75 mg doses resulted in a significant increase in C_max_ compared to no enzyme (Fig 3D).

### 3.4. Comparison to pancrelipase

#### 3.4.1. Total amino acids.

**Fig 4:**
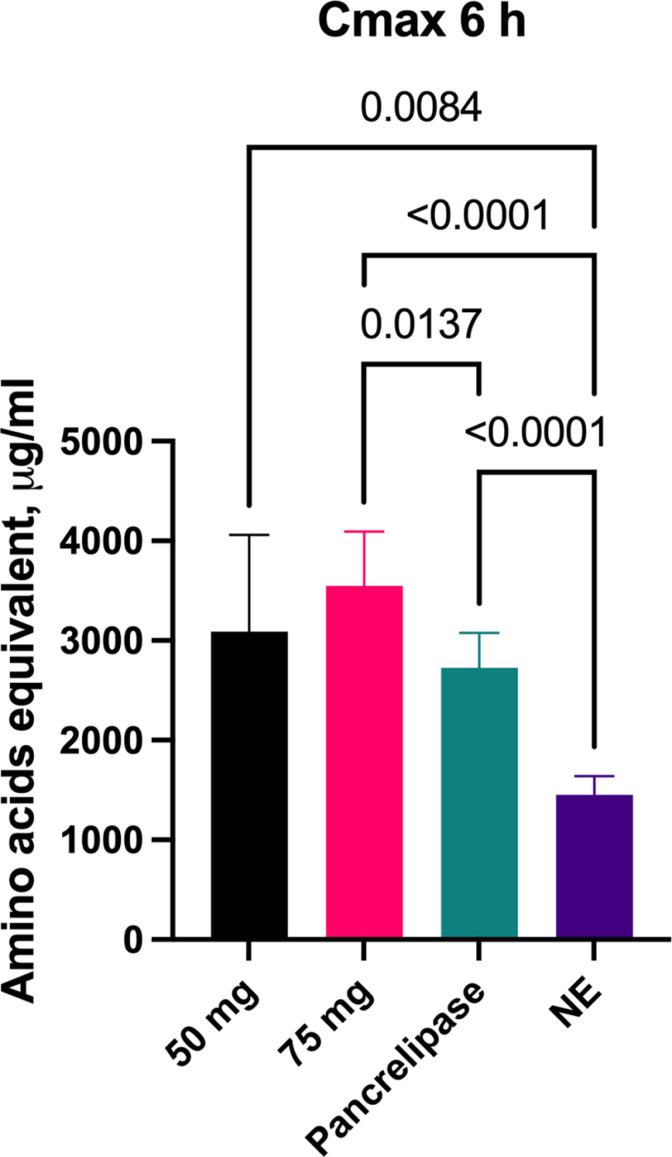
Comparison of aspergillus protease to porcine pancrelipase. Changes in plasma total amino acids concentrations over 6-hours(A), 6-hour area under the curve (AUC_6_) (B), mean concentration over 6-hours (C), C_max_ over 6 hours (D), following administration of either 50 mg protease, 75 mg protease or a 600 mg dose of pancrelipase, compared to no enzyme (NE). Data on AUC_6_ is presented as median ±IQR, other data is presented as mean ±SD. Differences were considered significant if p ≤ 0.05; differences were considered as a trend when p ≤ 0.1.

The change in absorbed plasma total amino acids over 6-hours was then studied following administration of either 50 mg or 75 mg of aspergillus protease or 600 mg pancrelipase, together with the whey substrate (Fig 4A). Compared to no enzyme, both aspergillus protease doses and pancrelipase significantly increase the AUC_6_ (Fig 4B), the mean plasma total amino acids concentration over 6-hours (Fig 4C), and Cmax (Fig 4D). Administration of 75 mg of aspergillus protease led to significantly higher AUC_6_ and Cmax when compared to pancrelipase (Fig 4C, D).

#### 3.4.2. *Peptide-derived amino acids.*

**Fig 5:**
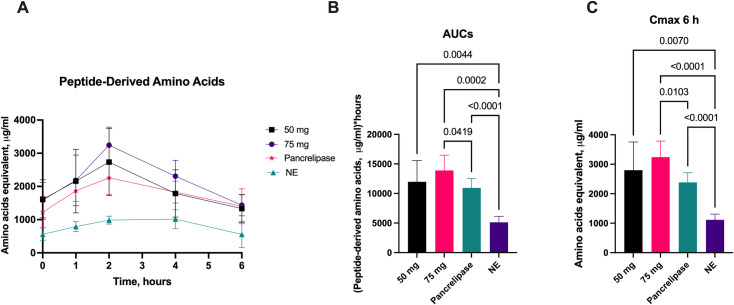
Comparison of aspergillus protease to porcine pancrelipase. Changes in plasma peptide-derived amino acids concentrations over 6-hours(A), 6-hour area under the curve (AUC_6_) (B), mean concentration over 6-hours (C), C_max_ over 6 hours (D), following administration of either 50 mg protease, 75 mg protease or a 600 mg dose of pancrelipase, compared to no enzyme (NE). Data on AUC_6_ is presented as median ±IQR, other data is presented as mean ±SD. Differences were considered significant if p ≤ 0.05; differences were considered as a trend when p ≤ 0.1.

The change in plasma peptide-derived amino acids over 6-hours following administration of either 50 mg or 75 mg of aspergillus protease or 600 mg pancrelipase, together with the whey substrate, is shown in Fig 5A. The AUC_6_ for plasma peptide-derived amino acids increased significantly following administration of aspergillus protease 50 mg, 75 mg, or pancrelipase when compared to no enzyme (Fig 5B). The AUC_6_ for peptide-derived amino acids after aspergillus protease increased by 133% for the 50 mg dose (p = 0.0044), by 171% for the 75 mg dose (p = 0.0002), and by 113% with 600 mg pancrelipase (p < 0.0001) when compared to no enzyme. Mean plasma peptide-derived amino acid concentration increased significantly compared to no enzyme for both aspergillus protease doses and for pancrelipase (Fig 5C). Likewise, Cmax increased significantly following administration of aspergillus protease 50 mg and 75 mg as well as with pancrelipase when compared to no enzyme (Fig 5D). Administration of 75 mg of aspergillus protease led to significantly higher AUC_6_ and Cmax when compared to pancrelipase (p = 0.0419 and 0.0103, respectively). (Fig 5C, D).

## 4. Discussion

As part of our modified ninhydrin methodology to quantify peptide-derived amino acids, we measured the concentration of absorbed free amino acids as equivalent amine groups in the plasma of EPI pigs. This amount was small and stayed relatively unchanged after a meal rich in whey protein and supplemented with proteases. This finding is consistent with direct measurement of amino acids in the human intestine after a protein load with bovine albumin [23], which shows that there are greater amounts of amino acids present as small peptides than in the free form in the gut lumen. Measurement of free amino acids in plasma is problematic as a measure of protease activity. Following provision of hydrolyzed proteins, there is preferential absorption of different types of amino acids[24], and plasma amino acid levels after an intact protein meal do not parallel the relative amino acid composition of the ingested food [25].

In contrast to the absorption of free amino acids, measurement of di- and tripeptides may be a more relevant outcome. These are structurally distinct molecules, with an estimated 400 dipeptides and 8000 tripeptides present after hydrolysis of dietary protein [26]. This diversity enables them to have complex physiological functions and may help with prevention of some chronic disease states [13,14]. Individual amino acids have one or two amine groups. Before hydrolysis, the simplest dipeptides possess a minimum of one amine group and would be recognized by ninhydrin and counted as one amino acid equivalent. After hydrolysis, dipeptides release at least two amine groups and tripeptides release at least three. The combination of all the amine group equivalents after hydrolysis is what we term total amino acids (free amino acids plus peptide-derived amino acids). We developed an algorithm, which is needed when measuring mixtures of all the products of dietary proteolysis with ninhydrin. This differs from optimization used to assess pure amino acid mixtures [27]. Using our formula for subtracting the free amino acids from the total amino acids, we found significant increases in post-prandial plasma peptide-derived amino acid concentration, AUC_6_, concentration over 6h and C_max_ when aspergillus protease is compared to no enzyme.

All the parameters measured for plasma peptide-derived amino acids (AUC_6,_ concentration, and Cmax) increased significantly following administration of either aspergillus protease or pancrelipase when compared to no enzyme. Notably, administration of 75 mg of aspergillus protease led to significantly higher AUC_6_ and Cmax when compared to 600 mg of pancrelipase. The amount of protease in pancrelipase is variable since it is a biologic extract. Requirements by the US Food and Drug Administration in the 2000’s narrowed the activity range allowed in each capsule [8]. If we presume the content is as labeled and the proportion of protease to lipase in pancrelipase is 3 to1 [22], the amount of protease provided in this experiment would be approximately 200 mg, which is more than twice the highest dose of aspergillus protease given in these experiments. The ability to assess protease dose is important because porcine extracts contain a higher proportion of protease per unit of lipase than is seen in humans [28,29], which is approximately 0.2 to 1. It is unknown what mechanism caused fibrosing colonopathy [30,31], a complication of high doses of PERT. PERT doses are expressed in lipase units, thus the amount of protease delivered with high doses of pancrelipase was not a focus of investigation. The development of a sensitive measure to study protease absorption can aid in the rational design of pancreatic enzyme replacement therapy for people with EPI.

This study has limitations. Pigs have different length and absorptive area of small intestine when compared to humans, potentially limiting the extrapolation of these results to people. In addition, porcine intestinal metabolism is more dependent on amino acids than is seen in humans [32]. The absorption of products of proteolysis depends not only on enzyme activity but also on the availability of transporters. There are age-related changes in PepT1 H^+^/peptide co- transporter expression along the gut in very young pigs, which express more PEP1 than adult pigs. However, this should not be a factor in pigs at the ages of those in this study.

In summary, we employed EPI pigs to study the activity of aspergillus protease given in combination with a novel lipase and fungal amylase, as well as commercially available pancrelipase. We were able to quantify protease activity when given with a meal and the presence of the two other major pancreatic digestive enzymes, which is a unique approach. Measurement of peptide-derived amino acids following a meal with dietary whey substrate differentiates the activity of aspergillus protease compared to no enzyme and the 75 mg dose was superior to pancrelipase. Studies in humans should be designed to support the evidence presented here in EPI pigs demonstrating that the whey substrate absorption challenge test (SACT) reflects the proteolytic activity of different doses of exogenous pancreatic proteases. The whey SACT has the potential to be a tool in the rational design of pancreatic enzyme replacement therapy for people with EPI.

## Supporting information

S1 File Raw data. (XLSX)
